# Synergistic optimisation of expression, folding, and secretion improves *E. coli* AppA phytase production in *Pichia pastoris*

**DOI:** 10.1186/s12934-020-01499-7

**Published:** 2021-01-07

**Authors:** Laura Navone, Thomas Vogl, Pawarisa Luangthongkam, Jo-Anne Blinco, Carlos Luna-Flores, Xiaojing Chen, Juhani von Hellens, Robert Speight

**Affiliations:** 1 Science and Engineering Faculty, Queensland University of Technology, Brisbane, QLD Australia; 2 Department of Computer Science and Applied Mathematics, Weizmann Institute of Science, Rehovot, Israel; 3 Bioproton Pty Ltd, Acacia Ridge, QLD Australia; 4 ARC Centre of Excellence in Synthetic Biology, Queensland University of Technology, Brisbane, QLD Australia

**Keywords:** Phytase, Strain engineering, Disulfide bond, Folding, Secretion

## Abstract

**Background:**

*Pichia pastoris* (*Komagataella phaffii*) is an important platform for heterologous protein production due to its growth to high cell density and outstanding secretory capabilities. Recent developments in synthetic biology have extended the toolbox for genetic engineering of *P. pastoris* to improve production strains. Yet, overloading the folding and secretion capacity of the cell by over-expression of recombinant proteins is still an issue and rational design of strains is critical to achieve cost-effective industrial manufacture. Several enzymes are commercially produced in *P. pastoris*, with phytases being one of the biggest on the global market. Phytases are ubiquitously used as a dietary supplement for swine and poultry to increase digestibility of phytic acid, the main form of phosphorous storage in grains.

**Results:**

Potential bottlenecks for expression of *E. coli* AppA phytase in *P. pastoris* were explored by applying bidirectional promoters (BDPs) to express AppA together with folding chaperones, disulfide bond isomerases, trafficking proteins and a cytosolic redox metabolism protein. Additionally, transcriptional studies were used to provide insights into the expression profile of BDPs. A flavoprotein encoded by *ERV2* that has not been characterised in *P. pastoris* was used to improve the expression of the phytase, indicating its role as an alternative pathway to *ERO1*. Subsequent AppA production increased by 2.90-fold compared to the expression from the state of the *AOX1* promoter.

**Discussion:**

The microbial production of important industrial enzymes in recombinant systems can be improved by applying newly available molecular tools. Overall, the work presented here on the optimisation of phytase production in *P. pastoris* contributes to the improved understanding of recombinant protein folding and secretion in this important yeast microbial production host.

## Background

Phytases (myo-inositol hexakisphosphate phosphohydrolases) are routinely added to livestock feed for the liberation of phosphate from phytate. The use of phytase reduces the addition of inorganic phosphate in poultry and swine diets and decreases the anti-nutritional effects of phytate [26, 34].

Phytases can be found in animals and plants but microbial phytases have been the most extensively studied and constitute all commercial formulations available in the livestock feed supplement market [16, 38]. Among commercial phytases, the *E. coli* AppA phytase shows high catalytic efficiency [37]. AppA phytase and derived variants have been produced in *Trichoderma reesei, Schizosaccharomyces pombe* and *P. pastoris* [16, 38]*.* There has been significant commercial work towards process optimisation to increase phytase production yields to achieve technical and economic manufacturing feasibility, but there is limited available information in the academic literature on *P. pastoris* strain engineering for phytase production. Previous investigations on the production of AppA phytase in *P. pastoris* include the expression under the strong constitutive promoter of the glyceraldehyde 3-phosphate dehydrogenase gene (*P*_*GAP*_) and methanol inducible alcohol oxidase 1 promoter (*P*_*AOX1*_) promoters [8, 9, 32, 38, 53, 56].

*P. pastoris* has become an important platform for heterologous protein production due to its growth to high cell density and high secretory capabilities, while secreting low amounts of endogenous proteins thereby simplifying down-stream processing. Significant development of molecular tools, including synthetic promoters for fine-tuning of expression, glyco-engineered strains and CRISPR/Cas9 technology, have driven the generation of highly secreting strains [65, 70].

Common approaches to improve heterologous protein production in *P. pastoris* include increasing gene copy number, use of strong inducible promoters and co-expression of chaperones to facilitate folding and secretion [13, 46, 63, 65–67]. For some enzymes, the presence of multiple disulfide bonds is challenging when striving for high and industrially compatible levels of expression due to the opportunity for incorrect bond formation and misfolding. AppA phytase has four disulfide bonds with one being non-consecutive, which means that the cysteines forming the pair are not consecutive in the protein sequence and have an increased chance of bonding between incorrect cysteine side chains [39]. Co-expressing suitable chaperone/s in a timely manner with the heterologous protein can therefore aid correct disulfide bond formation and rearrangement leading to correctly folded and active enzyme with reduced accumulation of mis-folded proteins.

When the accumulation of misfolded proteins exceeds the ER capabilities, the unfolded protein response (UPR) is activated through the splicing of HAC1 mRNA. HAC1 is the master regulator of the UPR which leads to transcription of a cascade of genes involved in protein folding and ER-associated protein degradation pathway (ERAD) [2].

Formation of disulfide bonds in the ER is possible due to the local oxidising environment. Proteins undergoing folding form disulfide bonds through dithiol-disulfide exchange with the oxidised form of the thioredoxin-like protein disulfide isomerase (PDI) [20]. PDI also catalyses the isomerisation of disulfide bonds [20, 30, 35, 36, 49].

Several engineering strategies can be applied at different points of the pathway to circumvent limitations for efficient recombinant protein production. Co-expression of folding and secretion chaperones has been successful in many cases but optimisation requires the simultaneous expression fine-tuning of multiple proteins to avoid cellular stress and undesirable mutations that reduce production [63, 64, 66, 67]. Co-expression of the gene of interest and folding helper proteins frequently necessitates fine-tuning of the ratio of the proteins to each other, their overall amounts (to avoid overburdening the cellular machinery), and the timing of expression (*i.e.* it may be beneficial to express a folding helper protein prior to its protein folding target).

Recently developed bidirectional promoters (BDPs) are particularly useful to achieve co-expression of target enzymes and chaperones that assist expression [63, 66, 67]. BDPs can initiate transcription on opposite sides and present diverse expression strengths, ratios, and regulatory profiles useful for optimisation of multiple gene expression. BDPs available for *P. pastoris* are not limited to methanol induction, but also offer constitute and derepressed regulatory profiles, which can be beneficial for scale up and industrial production. In this work, we used BDPs to co-express several chaperones and to increase production capability of *E. coli* AppA phytase in *P. pastoris,* surpassing expression from the commonly used *P*_*AOX1*_ by nearly three-fold*.*

## Methods

### Strains and growth conditions

The strain used in this study was the *P. pastoris* BG11 strain (derivative of *P. pastoris* BG10 strain, *ΔAOX1* (mutS—methanol utilization slow) from ATUM Inc. (Newark, California, USA). α-Select Silver efficiency competent *E. coli* strain (Bioline, Australia) was used for cloning. Cultivations were conducted in Luria Broth (LB) media for *E. coli* and yeast cultures were either grown in YPD medium (1% w/v yeast extract, 2% w/v peptone and 2% w/v glucose), buffered minimal dextrose (BMD) medium (1.34% Yeast Nitrogen Base YNB, 4 × 10^−5^% biotin, 200 mM potassium phosphate buffer pH 6.0 and 2% glucose), buffered minimal methanol (BMM) medium (1.34% YNB, 4 × 10^−5^% biotin, 200 mM potassium phosphate buffer pH 6.0) with 1% methanol (BMM2) or 5% methanol (BMM10). Antibiotic Zeocin (Invitrogen) was added to the media when required at a final concentration of 25 µg/mL for *E. coli* or 100 µg/mL for *P. pastoris* cultivations. Small scale cultivations of *P. pastoris* were performed using deep-well-plates (DWP) and induction protocols reported previously [69].

### Cloning and transformation of Pichia pastoris

The vector used for cloning and integration into *P. pastoris* genome was pD912 from ATUM Inc., which is based on the pPpT4S vector family [43]. Monodirectional promoter (MDP) sequences *P*_*AOX1*_, glyceraldehydes-3-phosphate dehydrogenase (*P*_*GAP*_), dihydroxyacetone synthase promoter (*P*_*DAS1*_), catalase 1 (*P*_*CAT1*_), and the orthologous formate dehydrogenase promoter from *Hansenula polymorpha* (*P*_*HpFMD*_) [62] were amplified with 50 bp overlapping sequences at 5′ and 3′ for Gibson assembly cloning into pD912 derived plasmids [11, 27, 29, 68]. BDP sequences *P*_*DAS1/2,*_, *P*_*HpFMD-HpMOX*_ and *P*_*AOX1-CAT1*_ were obtained for Bisy e.U. and cloned into pD912 derived plasmids following the strategy outlined by [63, 66, 67]. The AppA *E. coli* phytase gene (accession number X05471.1) was codon optimised by ATUM Inc. and expressed in the direction of the first promoter region indicated in the BDP name, using the α-mating factor secretion signal from *S. cerevisiae* [7]. The genes *HAC1* (spliced version) [25], *PDI* (accession number ACF17572.1), *ERV2* (accession number ANZ73509.1), *MPDI* (accession number XP_002489466), *ERO1* (accession number ANZ74048.1), *KAR2* (accession number ANZ77450.1), *GPX1* (accession number BAH57503.1), *SEC1* (accession number CAY67514.1), *SLY1* (accession number CCA40908.1), *EUG1* from *S. cerevisiae* (accession number XP_446874) were ordered as gBlocks from Integrated DNA Technologies (IDT) with 50 bp overlapping sequences at 5′ and 3′ for Gibson assembly cloning into pD912 derived plasmids. Chaperones were expressed in the direction of the second promoter region indicated in the BDP name (Additional file 1). *P. pastoris* cells were transformed with *Swa*I linearised plasmids following a condensed standard protocol [40] with up to 5 µg of total DNA. After the transformation, screening of transformants was performed in DWP as described previously [69].

### Phytase expression

AppA phytase expression was conducted in 250 mL baffled shake flasks following standard expression conditions at 28˚C, 250 rpm. The culture was grown in 50 mL of BMD1 for 65 h following methanol induction with BMM10 and consecutive additions of pure methanol 1% final concentration until harvest at 132 h. The protein concentration in the culture supernatant was determined using the Bradford method [5].

### Genomic DNA extraction and gene copy number determination

Genomic DNA was extracted using ISOLATE II Genomic DNA kit (Bioline Pty Ltd.; Alexandria, Australia) following the standard protocol with minor modifications as described previously [63, 66, 67]. Gene copy number determination was performed as described previously [6] using Bio-Rad QX200 Droplet Digital PCR (ddPCR). Genomic DNA was digested with *Sph*I restriction enzyme (New England Biolabs) and 0.5 ng of digested DNA of each sample was added to the reaction mixture. For DNA amplification of the *AppA* phytase gene a set of primers (Fwd 5′-TTGTCCTCAATCCGGTCAAG-3′ and Rev 5′-AGGGTTAAACAACGGATCGG-3′) and a 5′ hexachloro-fluorescein (HEX) dye-labelled probe were designed (5′-HEX-TTGGCTCCAGACTGTGCTATCACTGT- IB®FQ- 3′). The arginosuccinate lyase (*ARG4*) gene was used as reference gene and a set of primers (Fwd 5′-TGCGGTTGTATGTCAGAGAC-3′ and Rev 5′-GGTTGAGCTCTTTGCAAGTG-3′) and 5′ 6-fluorescein amidite (6-FAM) dye-labelled probe were designed (5′-FAM-TGGCTGACTATCTGAAGCAGTTCATTCA- IB®FQ- 3′) [1]. The probes were also labelled with Iowa Black® FQ at the 3′ terminus. All primers and probes were synthetised by IDT. Each PCR was performed in a 20 µL volume containing 12.5 µL of ddPCR Supermix for Probes (Bio-rad), 900 nM of each primer, 250 nM of each probe, 0.5 ng of digested genomic DNA and the required amount of MilliQ water. Droplets were generated using the Droplet Generator (Bio-Rad) and transferred into a 96-well plate for amplification. Thermal cycle conditions were 95˚C (10 min), 95˚C (30 s) and 58˚C (1 min) for 40 cycles, 98˚C (10 min). Droplet detection was carried out using QuantaSoft software (Bio-Rad) and the gene copy number was calculated for each sample in triplicate.

### RNA extraction and transcriptional studies

RNA was extracted using SV Total RNA Isolation System (Promega) following the standard protocol with minor modifications. Cell pellets of 10 OD_600_ units were resuspended in 1 M sorbitol, 0.1 M EDTA pH 7.4 and 0.1% β-mercaptoethanol. For cell lysis 175 µL of RNA Lysis Buffer was added and the suspension transferred to a microcentrifuge tube filled with RNAase-free glass beads. Cells were then vortex mixed for 20 min, centrifuged at maximum speed and the supernatant transferred to a new microcentrifuge tube. The extraction was continued according to the standard protocol. Absolute quantification was performed using One-Step RT-ddPCR Advanced Kit for Probes (Bio-Rad) for reverse transcription and PCR amplification following the manufacture’s specifications. For amplification of *AppA* and *ARG4* cDNA the same set of primers and probes described above were used. For *pdi* cDNA a set of primers (Fwd 5′-GCAGGAGTCGAGTCGCTAGTGT-3′ and Rev 5′-TTGCCTCGGCGATTGTGTCG-3′) and a 5′-hexachloro-fluorescein (HEX) dye-labelled probe were designed (5′-HEX-TTCTCTCTGAGAGTACCGGCAACTCCG-3′). The probe was also labelled with Iowa Black® FQ at the 3′ terminus. All primers and probes were synthetised by IDT, Australia. Each PCR was performed in a 20 µL reaction volume containing 5 µL of Supermix (Bio-Rad), 2 µL of reverse transcriptase (Bio-rad), 1 µL of 300 mM DTT, 900 nM of each primer, 250 nM of *ARG4*-HEX, *AppA*-FAM probe or 125 nM of *PDI*-HEX probe, 0.3 ng of RNA and the required amount of MilliQ water. Droplets were generated using the Droplet Generator (Bio-Rad) and transferred into a 96-well plate for retrotranscription and amplification. Thermal cycle conditions were 60˚C (60 min), 95˚C (10 min), 95˚C (30 s) and 58˚C (1 min) for 40 cycles, 98˚C (10 min). Droplet detection was carried out using QuantaSoft software (Bio-Rad) and absolute quantification determination was calculated for each sample in triplicate.

### Enzyme activity quantification

Acid phosphatase activity was assayed to measure phytase expression levels using a assay based on the substrate *para*-nitrophenylphosphate (p-NPP) (Sigma) at an initial concentration of 5 mM in the assay [17]. Briefly, 10 µL of five enzyme dilutions were incubated with 90 µL of p-NPP substrate in 250 mM sodium acetate buffer pH 5.5 for 15 min at 37 °C. The reaction was stopped by the addition of 10 µL of 1 M NaOH. The released *para*-nitrophenol was measured at 410 nm after 10 min incubation at room temperature. Reactions were conducted in triplicate in 96-well plates.

One unit of acid phosphatase activity was defined as the amount of enzyme catalysing the formation of 1 µmole of *para*-nitrophenol per minute under the assay conditions.

## Results

Transcriptional optimisation of *AppA* was conducted using methanol inducible, de-repressed, and constitutive MDPs from *P. pastoris* and *H. polymorpha*. Several BDPs were then tested for co-expression of the phytase together with folding and secretion chaperones, comparing phytase production yields with the commonly used *P*_*AOX1*_.

The co-expression of *AppA* with chaperones for disulfide bond formation, vesicle transport between organelles and ROS detoxification was conducted to address potential bottlenecks for AppA secretion in *P. pastoris*. Transcriptional analysis using droplet digital PCR was applied to follow the expression of *AppA* phytase and *PDI* transcripts during production, bringing new insights into the BDP expression profile.

### Transcriptional optimization of AppA phytase expression

**Fig. 1:**
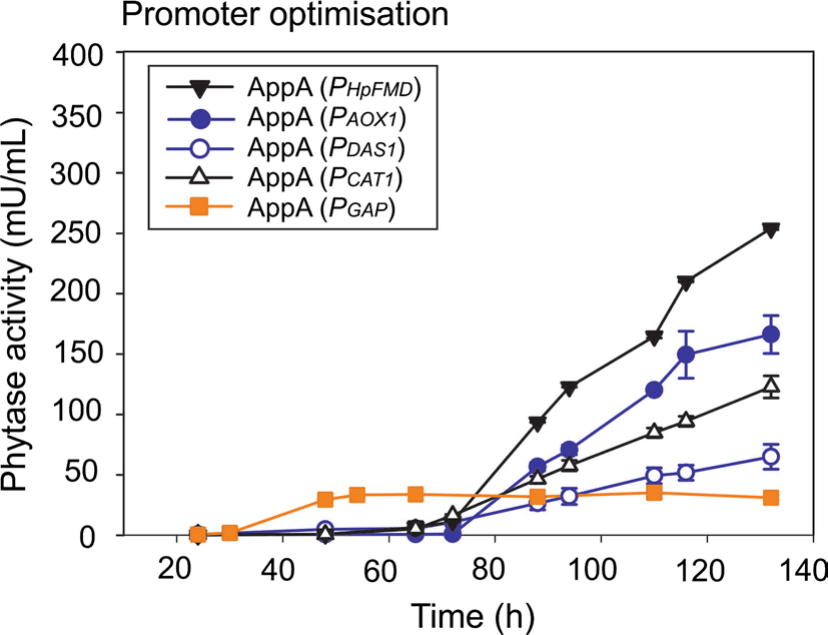
AppA expression from *P*_*HpFMD*_ surpasses *P. pastoris* endogenous MDPs (*P*_*AOX1*_, *P*_*DAS1*_, *P*_*CAT1*_ and *P*_*GAP*_). Phytase expression was determined by the p-NPP assay. All strains except AppA (*P*_*GAP*_) were induced with methanol at 65 h and consecutive additions until 132 h. Data are represented as mean values ± standard deviation (n = 3)

The *AppA* gene from *E. coli* was expressed under the control of the commonly used methanol inducible *P. pastoris* MDPs *P*_*AOX1*_, *P*_*DAS1*_ and *P*_*CAT1*_, the methanol inducible *P*_*HpFMD*_ from *H. polymorpha* [62] and the constitutive *P. pastoris* promoter *P*_*GAP*_ (Fig. 1). No difference in growth rate was observed between the strains (data not shown). The highest production of AppA phytase was observed for AppA (*P*_*HpFMD*_) strain showing a 1.53 ± 0.01-fold increase after 132 h compared to AppA (*P*_*AOX1*_) (Table 1). Expression from the constitutive promoter *P*_*GAP*_ showed very low production of AppA phytase under the conditions tested (Fig. 1).

**Table 1:** Fold change of AppA phytase production relative to MDP *P*_*AOX1*_, *P*_*HpFMD*_ or BDP *P*_*HpFMD-HpMOX*_

Strain	Fold change relative to AppA (*P*_*AOX1*_)	Fold change relative to AppA (*P*_*HpFMD*_)	Fold change relative to AppA-PDI (*P*_*HpFMD-HpMOX*_)
AppA (*P*_*HpFMD*_)	1.53 ± 0.01*	–	–
AppA-HAC1 (*P*_*HpFMD-HpMOX*_)	2.08 ± 0.07*	1.36 ± 0.05**	–
AppA-HAC1 (*P*_*AOX1-CAT1*_)	1.31 ± 0.09*	0.86 ± 0.06**	–
AppA-HAC1 (*P*_*DAS1/2*_)	0.54 ± 0.03*	0.35 ± 0.02**	–
AppA-PDI (*P*_*HpFMD-HpMOX*_)	2.14 ± 0.03*	1.40 ± 0.02**	–
AppA-PDI (*P*_*AOX1-CAT1*_)	1.71 ± 0.07*	1.12 ± 0.05**	0.80 ± 0.03***
AppA-PDI (*P*_*DAS1/2*_)	1.53 ± 0.50*	1.00 ± 0.13	0.71 ± 0.09***
AppA-MPDI (*P*_*HpFMD-HpMOX*_)	0.68 ± 0.16*	0.44 ± 0.10**	–
AppA-EUG1 (*P*_*HpFMD-HpMOX*_)	1.37 ± 0.02*	0.90 ± 0.01**	–
AppA-SEC1 (*P*_*HpFMD-HpMOX*_)	1.54 ± 0.04*	1.01 ± 0.02	–
AppA-SLY1 (*P*_*HpFMD-HpMOX*_)	1.63 ± 0.01*	1.06 ± 0.01	–
AppA-ERV2 (*P*_*HpFMD-HpMOX*_)	2.38 ± 0.03*	1.56 ± 0.02**	1.11 ± 0.01***
AppA-ERO1 (*P*_*HpFMD-HpMOX*_)	0.79 ± 0.11*	0.52 ± 0.07**	0.38 ± 0.05***
AppA-PDI-ERO1 (*P*_*GAP*_)	2.59 ± 0.12*	1.70 ± 0.08**	1.21 ± 0.06***
AppA-PDI-ERO1 (*P*_*CAT1*_)	2.33 ± 0.24*	1.52 ± 0.16**	1.09 ± 0.11
AppA-PDI-ERV2 (*P*_*GAP*_)	2.90 ± 0.08*	1.90 ± 0.05**	1.35 ± 0.04***
AppA-PDI-ERV2 (*P*_*CAT1*_)	2.42 ± 0.12*	1.59 ± 0.08**	1.13 ± 0.05
AppA-PDI-GPX1 (*P*_*GAP*_)	1.71 ± 0.01*	1.12 ± 0.01	0.80 ± 0.01***
AppA-PDI-GPX1 (*P*_*CAT1*_)	2.8 ± 0.31*	1.83 ± 0.20**	1.30 ± 0.14***
AppA-PDI-HAC1 (*P*_*CAT1*_)	2.26 ± 0.35*	1.48 ± 0.16**	1.06 ± 0.16
AppA-HAC1-PDI (*P*_*GAP*_)	1.72 ± 0.23*	1.13 ± 0.15	0.80 ± 0.11***
AppA-PDI-KAR2 (*P*_*GAP*_)	2.01 ± 0.01*	1.32 ± 0.01**	0.94 ± 0.02

^a^All strains have a single copy of the integrated cassette (CNV = 1) as determined by ddPCR

^*^Significantly different from AppA (*P*_*AOX1*_) (p value ˂ 0.05)

^**^Significantly different from AppA (*P*_*FMD*_) (p value ˂ 0.05)

^***^Significantly different from AppA-PDI (*P*_*HpFMD-HpMOX*_) (p value ˂ 0.05)

### AppA phytase co-expression with HAC1 and ER-associated disulfide bond formation chaperones using bidirectional promoters

**Fig. 2:**
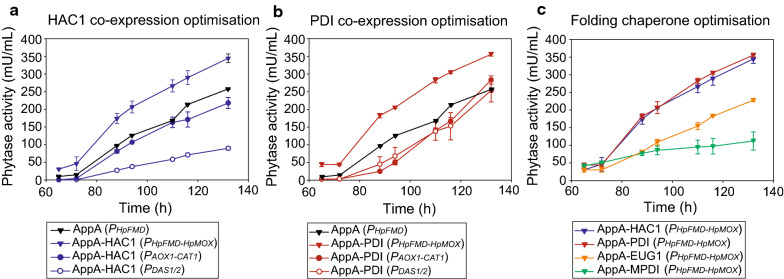
HAC1 or PDI co-expression from BDPs further increases phytase yields. **a** AppA co-expressed with HAC1 under *P*_*AOX1-CAT1*_, *P*_*DAS1/2*_ and *P*_*HpFMD-HpMOX*_. **b** AppA co-expressed with PDI under the BDPs *P*_*AOX1-CAT1,*_* P*_*DAS1/2*_ and *P*_*HpFMD-HpMOX.*_ **c** AppA co-expressed with MPDI and EUG1 under the BDP *P*_*HpFMD-HpMOX*_. The AppA-HAC1 (*P*_*HpFMD-HpMOX*_) and AppA-PDI (*P*_*HpFMD-HpMOX*_) strains were included for comparison. Phytase expression was determined by the pNPP assay after methanol induction. Data are represented as mean values ± standard deviation (n = 3)

AppA phytase was then co-expressed with the unfolded protein response transcriptional regulator HAC1 under the methanol inducible BDPs *P*_*AOX1-CAT1*_, *P*_*HpFMD-HpMOX*_ and *P*_*DAS1/2*_ [63, 66, 67]. Previous reports have showed that co-expression with HAC1 increases expression of several recombinant proteins [3, 22, 25, 59, 59, 60, 60]. Figure 2a shows a comparison between co-expression of HAC1 with different BDPs. Expression of AppA phytase showed 1.36 ± 0.05-fold increase after 132 h when co-expressing HAC1 compared to the AppA (*P*_*FMD*_), and 2.08 ± 0.01-fold increase when compared to the expression from the MDP *P*_*AOX1*_ (Table 1). In the case of AppA-HAC1 (*P*_*AOX1-CAT1*_) and AppA-HAC1 (*P*_*DAS1/2*_) strains, the co-expression of HAC1 appeared to improve the production of the phytase by a similar relative ratio compared to the corresponding MDP expression strain AppA (*P*_*AOX1*_) and AppA (*P*_*DAS1*_). AppA-HAC1 (*P*_*AOX1-CAT1*_) showed a 1.31 ± 0.01-fold increase compared to AppA (*P*_*AOX1*_) (Table 1), while phytase production in AppA-HAC1 (*P*_*DAS1/2*_) improved 1.38 ± 0.07-fold compared to AppA (*P*_*DAS1*_). The results show that the effect of HAC1 on phytase production is independent of the promoter driving *HAC1* gene expression, (*P*_*HpMOX*_, *P*_*CAT1*_ or *P*_*DAS2*_) leading to a ~ 30% increased yield in all cases, suggesting that the threshold of HAC1 regulator required to activate UPR is reached when expressed from any of the promoters.

One of the disulfide bonds is AppA phytase is a non-consecutive bond (C155-C430) which increases the opportunity for mispairing [39]. In *E. coli* the correct formation of this bond depends on DsbC, a protein-disulfide isomerase capable of correcting incorrectly formed bonds [4]. For enhancement of AppA phytase production in *P. pastoris,* the overexpression of a protein-disulfide isomerase might be advantageous. The co-expression of PDI using the *P*_*HpFMD-HpMOX*_ BDP improved production of AppA phytase by 2.14 ± 0.03-fold compared to AppA (*P*_*AOX1*_) and 1.40 ± 0.02-fold compared to AppA (*P*_*HpFMD*_), showing very similar results to AppA-HAC1 (*P*_*HpFMD-HpMOX*_) strain (Table 1). Since the *PDI* gene is regulated by the UPR, the similarity in phytase production between AppA-PDI (*P*_*HpFMD-HpMOX*_) and AppA-HAC1 (*P*_*HpFMD-HpMOX*_) strains could indicate an activation of *PDI* gene transcription by HAC1 in the AppA-HAC1 (*P*_*HpFMD-HpMOX*_) strain, hinting towards a dominant effect of PDI in the HAC1 co-expression.

The co-expression of recombinant proteins and chaperones requires fine tuning, as both their relative ratios and cumulative amounts are important for the final expression levels [63, 66, 67]. The co-expression of AppA with PDI under BDPs with alternative expression profiles could result advantageous for final phytase yields by balancing protein amounts relative to each other. Based on this idea, strains AppA-PDI (*P*_*AOX1-CAT1*_) and AppA-PDI (P_DAS1/2_) were also constructed. As shown in Fig. 2b, the co-expression of AppA and PDI from the *P*_*AOX1-CAT1*_ or P_DAS1/2_ did not improve production compared to the expression from *P*_*HpFMD-HpMOX*_. In fact, both strains showed decreased phytase production. Nevertheless, the co-expression of PDI was still favourable for AppA phytase production when compared with MDP AppA (*P*_*AOX1*_) and AppA (*P*_*DAS1*_) strains. The fold change observed compared to AppA-PDI (*P*_*HpFMD-HpMOX*_) was 1.71 ± 0.07 and 3.93 ± 0.5 for AppA-PDI (*P*_*AOX1-CAT1*_) and AppA-PDI (P_DAS1/2_), respectively.

From the BDPs tested by co-expression of HAC1 or PDI, *P*_*HpFMD-HpMOX*_ showed the highest phytase yields and corresponding strains were constructed using this BDP for co-expression of different chaperones (Fig. 2a, b). These results demonstrate for the first time that orthologous promoters can be fused into MDPs maintaining their beneficial properties related to strength and regulation, thereby extending the applicability of this concept beyond MDPs [62].

The co-expression of PDI has been shown previously to increase the expression of recombinant proteins in *P. pastoris,* however PDI homologues have not yet been tested for co-expression of heterologous proteins in this yeast. Considering that different homologues are proposed to interact and mediate thiol exchange with specific protein targets, we tested two PDI homologues for co-expression with *AppA* phytase (Fig. 2c) [57]. MPDI from *P. pastoris* presents high similarity to PDI while EUG1 from *S. cerevisiae* does not have a homologue in *P. pastoris* [54, 55]. Co-expression of MPDI had a marked detrimental effect on phytase production, while EUG1 improved production compared to AppA (*P*_*AOX1*_) but was less than AppA (*P*_*HpFMD*_) (Fig. 2c, Table 1), suggesting a possible overloading of the protein synthesis cellular machinery.

### Characterisation of bidirectional promoter ***P***_***HpFMD-HpMOX***_ expression profile

**Fig. 3:**
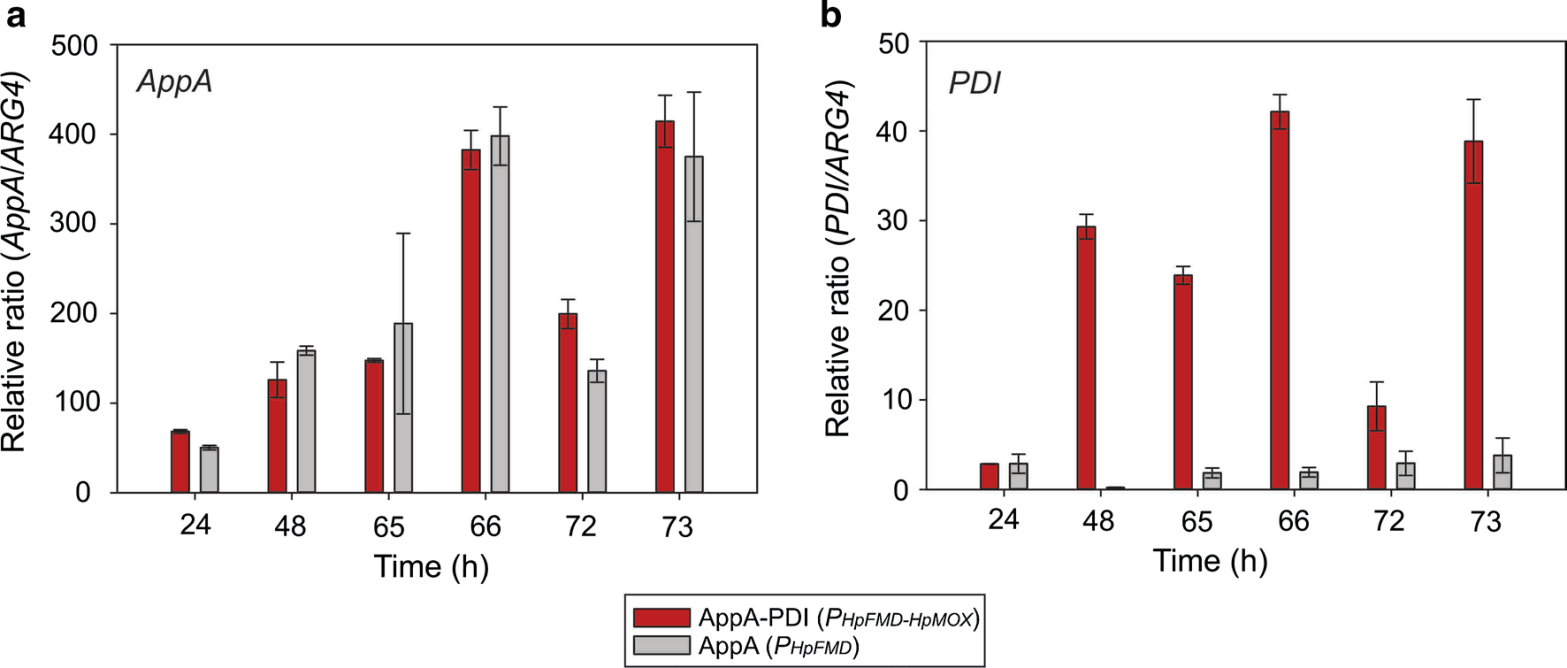
Transcriptional studies of *AppA* and *PDI genes* during phytase production. **a** Relative transcription of the *AppA* gene and **b** the *PDI* gene at 24, 48, 65, 66, 72 and 73 h. Methanol additions were performed at 65 and 72 h. *ARG4* was used as reference gene. Data are represented as mean values ± standard deviation (n = 3)

Transcriptional analysis of *AppA* and *PDI* genes were conducted to study the expression profile from the BDP *P*_*HpFMD-HpMOX*_ during phytase production and to verify the expression of *PDI* (Fig. 3). We used the AppA-PDI (*P*_*HpFMD-HpMOX*_) strain co-expressing AppA from *P*_*HpFMD*_, PDI from *P*_*HpMOX*_, because of the high phytase yields obtained. Since the total expression of *PDI* in the AppA-PDI (*P*_*HpFMD-HpMOX*_) strain is derived from the native *PDI* gene and the integrated *PDI* gene, the AppA (*P*_*HpFMD*_) strain was included for comparison where only expression from the native *PDI* gene was present. Transcriptional expression was analysed during glucose depletion (24, 48 h), immediately before methanol addition (65 and 72 h) and 1 h after methanol addition (66 and 73 h). The *AppA* phytase and *PDI* transcript levels relative to the *ARG4* reference gene were consistent and reached similar levels after each methanol addition (Fig. 3). Transcription of the *AppA* gene reached similar levels in the AppA (*P*_*HpFMD*_) and AppA-PDI (*P*_*HpFMD-HpMOX*_) strains showing that the final production levels were associated with the co-expression of *PDI*. Both *AppA* and *PDI* transcription from *P*_*HpFMD-HpMOX*_ started during glucose depletion sometime on or before 24 h. Transcription of both genes kept increasing up to 65 h, most likely due to the continuing drop in glucose levels and enhanced de-repression of the promoters. Transcriptional analysis of *PDI* in the AppA (*P*_*HpFMD*_) strain indicated the level of transcription associated with the native *PDI* gene alone. The transcription of *PDI* reached up to 3.8 times that of the *ARG4* gene, consistent with the increased transcription of the *AppA* phytase during methanol addition (Fig. 3b). The cumulative transcription of the isomerase in AppA-PDI (*P*_*HpFMD-HpMOX*_), corresponding to the native and extra copy of *PDI*, showed an approximate tenfold increase after each methanol addition compared to the expression of the native gene in AppA (*P*_*HpFMD*_).

Assuming the stability of *AppA* and *PDI* mRNAs to be similar, a marked difference in the strength of the *P*_*HpFMD*_ and *P*_*HpMOX*_ promoters was observed when comparing the transcription of *AppA* and *PDI*. Phytase expression from *P*_*HpFMD*_ reached a relative ratio of ~ 400 (Fig. 3a) while *PDI* expression from *P*_*HpMOX*_ showed a maximum relative ratio of ~ 40 after 1 h methanol additions (66 and 73 h) (Fig. 3b).

### Co-expression of AppA phytase with trafficking associated proteins

Protein trafficking pathways can represent a bottleneck for heterologous protein production in *P. pastoris* [15, 31]. Two strains co-expressing AppA phytase and proteins involved in trafficking were constructed, AppA-SEC1 (*P*_*HpFMD-HpMOX*_) and AppA-SLY1 (*P*_*HpFMD-HpMOX*_). SLY1 regulates ER-Golgi trafficking and SEC1 interacts with vesicle trafficking between the Golgi and cell membrane [28, 41]. No marked improvement of phytase production was observed for either AppA-SEC1 (*P*_*HpFMD-HpMOX*_) or AppA-SLY1 (*P*_*HpFMD-HpMOX*_) strains when compared to the AppA (*P*_*HpFMD*_) strain (Additional file 2: Fig. S1, Table 1). The results suggest that either vesicular transport of the phytase is not a bottleneck, or that the co-overexpression of SEC1 or SLY1 proteins is not enough to alleviate the burden on the secretion pathway. The co-expression of other transport associated proteins or the combination with PDI might be required to improve AppA production.

### AppA phytase production is improved when co-expressing with PDI and ERV2, ERO1 or GPX1

**Fig. 4:**
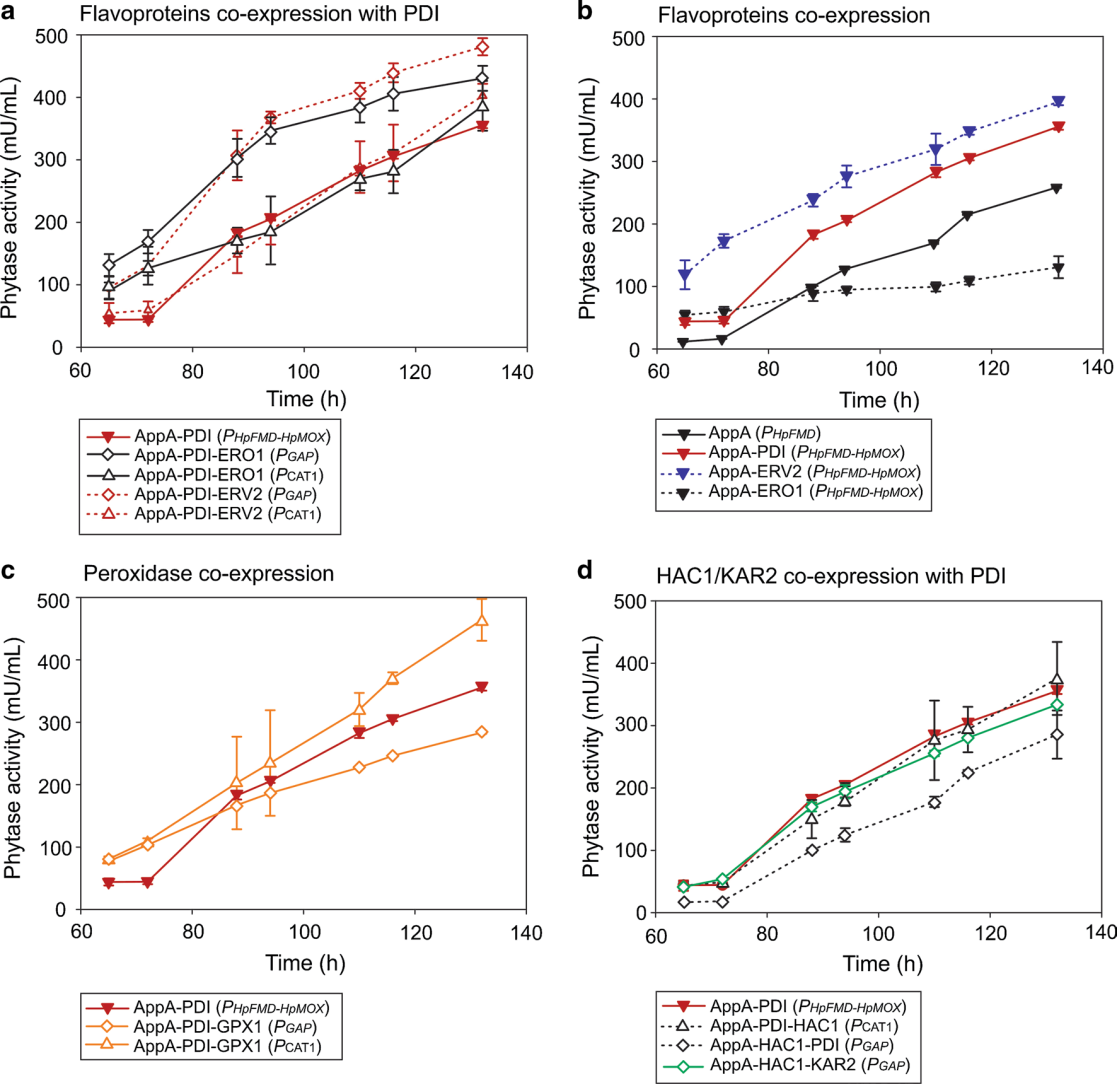
ERO1, ERV2 and GPX1 co-expression further improves phytase yields while co-expression of HAC1 or KAR2 does not show improvement. **a** AppA co-expression with PDI and ERO1 or ERV2. **b** AppA co-expression with ERO1 or ERV2. **c** AppA co-expression with GPX1. **d** AppA co-expression with HAC1 or KAR2. Phytase production was determined by the p-NPP assay after methanol induction. The AppA-PDI (*P*_*HpFMD-HpMOX*_) strain was included for comparison. Data are represented as mean values ± standard deviation (n = 3)

**Fig. 5:**
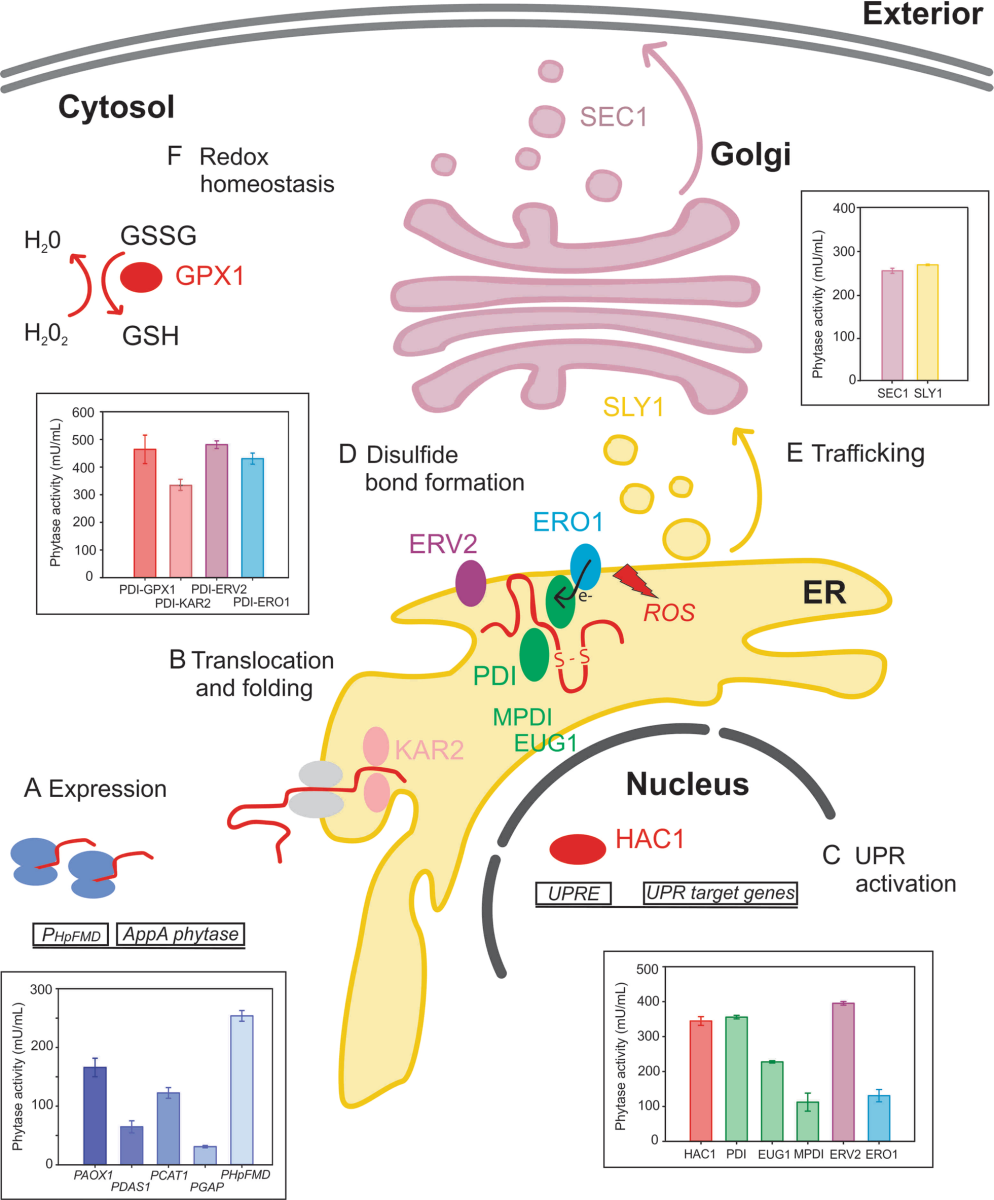
Overview of the strain engineering strategies applied in this work and secretory pathway bottlenecks explored. Column graphs represent AppA phytase activity (mU/mL) after 132 h of cultivation. Strains names are abbreviated for simplicity: AppA (*P*_*AOX1*_) as *P*_*AOX1*_, AppA (*P*_*DAS1*_) as *P*_*DAS1*_, AppA (*P*_*CAT1*_) as *P*_*CAT1*_, AppA (*P*_*GAP*_) as *P*_*GAP*_, AppA (*P*_*HpFMD*_) as *P*_*HpFMD*_, AppA-HAC1 (*P*_*HpFMD-HpMOX*_) as HAC1, AppA-PDI (*P*_*HpFMD-HpMOX*_) as PDI, AppA-EUG1 (*P*_*HpFMD-HpMOX*_) as EUG1, AppA-MPDI (*P*_*HpFMD-HpMOX*_) as MPDI, AppA-ERV2 (*P*_*HpFMD-HpMOX*_) as ERV2, AppA-ERO1 (*P*_*HpFMD-HpMOX*_) as ERO1, AppA-SEC1 (*P*_*HpFMD-HpMOX*_) as SEC1, AppA-SLY1 (*P*_*HpFMD-HpMOX*_) as SLY1, AppA-PDI-KAR2 (*P*_*GAP*_) as PDI-KAR2, AppA-PDI-GPX1 (*P*_*CAT1*_) as PDI-GPX1, AppA-PDI-ERV2 (*P*_*GAP*_) as PDI-ERV2 and AppA-PDI-ERO1 (*P*_*GAP*_) as PDI-ERO1

Due to the requirement for restoring oxidised PDI and the potential related limitation in the production of phytase in the AppA-PDI (*P*_*HpFMD-HpMOX*_) strain, four new strains co-expressing flavoproteins ERO1 and ERV2 were constructed [20, 50]. Two different promoters (*P*_*GAP*_ and *P*_*CAT1*_) were tested for the expression of ERO1 and ERV2 due to potential problems arising from cumulative amounts of expressed proteins. Furthermore, timing of expression may also play an important role for improved yields hence the constitutive *P*_*GAP*_ and methanol inducible *P*_*CAT1*_ were tested for expression of the second chaperone. The strains were named AppA-PDI-ERO1 (*P*_*GAP*_) and AppA-PDI-ERV2 (*P*_*GAP*_), AppA-PDI-ERO1 (*P*_*CAT1*_) and AppA-PDI-ERV2 (*P*_*CAT1*_) when ERO1 and ERV2 were expressed under the control of *P*_*GAP*_ or *P*_*CAT1*_, respectively. Figure 4a shows and improvement in phytase production for AppA-PDI-ERO1 (*P*_*GAP*_) and AppA-PDI-ERV2 (*P*_*GAP*_), 1.21 ± 0.06 and 1.35 ± 0.04-fold increase from AppA-PDI (*P*_*HpFMD-HpMOX*_), respectively. On the other hand, the strains AppA-PDI-ERO1 (*P*_*CAT1*_) and AppA-PDI-ERV2 (*P*_*CAT1*_) did not show a marked improvement when compared to AppA-PDI (*P*_*HpFMD-HpMOX*_), with a 1.09 ± 0.11 and 1.13 ± 0.05-fold change, respectively. The expression of the flavoproteins ERO1 and ERV2 under the control of the constitutive promoter *P*_*GAP*_ appeared to be more suitable compared to the expression from *P*_*CAT1*_. The effect could be related to the earlier expression of the flavoproteins from *P*_*GAP*_ before production of PDI and AppA by glucose de-repression and methanol induction [29, 42, 71]. Expression of ERV2 or ERO1 under *P*_*GAP*_ could alleviate stress on the cell machinery by production of the proteins during growth and not during methanol induction when PDI and AppA phytase were being produced (Fig. 5).

The improvement of phytase production in AppA-PDI-ERO1 (*P*_*GAP*_) and AppA-PDI-ERV2 (*P*_*GAP*_) strains could also be related to the co-expression of the flavoproteins, independently of PDI overexpression. AppA-ERO1 (*P*_*HpFMD-HpMOX*_) and AppA-ERV2 (*P*_*HpFMD-HpMOX*_) strains were constructed to test this hypothesis. As shown in Fig. 4b phytase production in the AppA-ERV2 (*P*_*HpFMD-HpMOX*_) strain was very similar (1.11 ± 0.01-fold change) to AppA-PDI (*P*_*HpFMD-HpMOX*_). On the other hand, the decrease in phytase production from the AppA-ERO1 (*P*_*HpFMD-HpMOX*_) strain compared to AppA-PDI (*P*_*HpFMD-HpMOX*_) (0.37 ± 0.05-fold change) could indicate a futile overload of the production machinery. Similar production of AppA phytase in AppA-ERV2 (*P*_*HpFMD-HpMOX*_) and AppA-PDI (*P*_*HpFMD-HpMOX*_) suggests that ERV2 flavoprotein might play a direct role in disulfide bond formation as previously proposed [24]. The results suggest that the improvement in AppA-PDI-ERO1 (*P*_*GAP*_) and AppA-PDI-ERV2 (*P*_*GAP*_) strains was most likely a synergistic effect of expressing PDI together with ERO1 or ERV2, and regeneration of oxidised PDI for further disulfide bonding in AppA phytase.

Overexpression of heterologous proteins with a high number of disulfide bonds can affect cellular redox homeostasis [14, 57]. To address a potential redox imbalance as a limitation in phytase production, two strains were constructed co-expressing the antioxidant enzyme GPX1. The strains were named AppA-PDI-GPX1 (*P*_*GAP*_) and AppA-PDI-GPX1 (*P*_*CAT1*_) when GPX1 was expressed under the control of the *P*_*GAP*_ or *P*_*CAT1*_, respectively. Contrary to the expression of ERO1 and ERV2 flavoproteins, production of AppA phytase was improved when GPX1 was expressed under *P*_*CAT1*_ (1.30 ± 0.14-fold change), and decreased under *P*_*GAP*_ expression (0.80 ± 0.01 fold change) compared to AppA-PDI (*P*_*HpFMD-HpMOX*_) strain (Fig. 4c). Since GPX1 is a peroxidase, expression during growth under *P*_*GAP*_ might create an unnecessary redox imbalance that affects phytase production. Importantly, co-expression of GPX1 did not affect growth of either strain (data not shown). The expression of GPX1 under *P*_*CAT1*_ appears to work better with the expression of the AppA phytase under methanol induction when the maintenance of redox homeostasis is required and not necessary during the growth phase.

Based on the initial increase in AppA phytase production when co-expressing HAC1 (Fig. 2a), two more strains were constructed to test for further improvement, AppA-PDI-HAC1 (*P*_*CAT1*_) and AppA-HAC1-PDI (*P*_*GAP*_). In the AppA-PDI-HAC1 (*P*_*CAT1*_) strain HAC1 was expressed from the *P*_*CAT1*_ and in AppA-HAC1-PDI (*P*_*GAP*_) strain, HAC1 was expressed from the BDP *P*_*HpFMD-HpMOX*_ and PDI is expressed from *P*_*GAP*_. The AppA-HAC1-PDI (*P*_*GAP*_) strain was designed to test if the effect on phytase production was similar when PDI was expressed under constitutive or inducible expression from *P*_*GAP*_ or *P*_*HpMOX*_ (in BDP *P*_*HpFMD-HpMOX*_), respectively. AppA-PDI-HAC1 (*P*_*CAT1*_) showed a similar yields to AppA-PDI (*P*_*HpFMD-HpMOX*_) (1.06 ± 0.16-fold change) suggesting that the co-expression of HAC1 under *P*_*CAT1*_ did not significantly contribute to AppA production in a PDI expressing strain background (Fig. 4d). These results may indicate that PDI and HAC1 lead to a similar, non-additive, beneficial effect. Furthermore, when HAC1 was expressed under the BDP *P*_*HpFMD-HpMOX*_ and PDI under *P*_*GAP*_, no marked improvement in phytase production was observed compared to AppA (*P*_*FMD*_) (1.13 ± 0.15-fold change) and the expression slightly decreased compared to AppA-PDI (*P*_*HpFMD-HpMOX*_) by 0.80 ± 0.11-fold (Fig. 4d). The expression of PDI under the inducible BDP *P*_*HpFMD-HpMOX*_ appeared to play an important role in the improvement of AppA production.

Over-expression of ER chaperone KAR2 has been shown to improve secretion of recombinant proteins [12, 23, 52, 72, 74], to test for improvement of AppA production, strain AppA-PDI-KAR2 (*P*_*GAP*_) was constructed. In this case, the expression of the KAR2 chaperone was only studied using *P*_*GAP*_. Results in Fig. 4d show that the co-expression of the chaperone did not further improve phytase production compared to the AppA-PDI (*P*_*HpFMD-HpMOX*_) strain (0.94 ± 0.02-fold change).

## Discussion

The development of *P. pastoris* as a production platform has made available a diverse toolbox for genetic improvement of strains. Using some of these tools, we have explored bottlenecks in folding and secretion of *E. coli* AppA phytase to gain a deeper understanding of *P. pastoris* as a production host. AppA phytase is widely used in animal feed to improve the digestibility of phytic acid. Extensive research has been conducted towards the improvement of the enzymatic properties of AppA as well as optimisation of fermentation processes [8, 9, 18, 21, 42, 44, 47, 53, 73]. Yet, fewer studies on strain engineering for enhancement of production yields have been conducted using *P. pastoris* as production host.

Transcriptional optimisation of *AppA* expression was conducted using the methanol inducible *P*_*AOX1*_, *P*_*DAS1*_, *P*_*CAT1*_ and *P*_*HpFMD*_, and the constitutive *P*_*GAP*_, obtaining highest phytase yields under the control of *P*_*HpFMD*_. These results confirms the applicability of the orthologous *FMD* promoter from *H. polymorpha* in *P. pastoris* also for secreted proteins, that are difficult to fold, whereas previous reports have focused mostly on intracellular recombinant proteins [62]. An investigation of consecutive checkpoints along the folding and secretion pathway was then conducted using BDPs (*P*_*AOX1-CAT1*_, *P*_*DAS1/2*_ and *P*_*HpFMD-HpMOX*_). Notably, among the BPDs studied, *P*_*HpFMD-HpMOX*_ showed the highest AppA production during co-expression with chaperones (Fig. 2a and b, Table 1). This shows that the two orthologous promoters from *H. polymorpha* (*P*_*HpFMD*_ and P_*HpMOX*_) can be combined into BDPs whilst maintaining their specific strength and regulation [62].

Synergistic effects of fusing different pairs of MDPs to each other were previously investigated [63, 66, 67]. For a few BDPs involving shortened MDPs (˂500 bp), where putative 5′ insulating ends were removed, the authors showed elevated expression or ‘transcriptional spill over’. However, nearly all full fusions, where the length of each MDP was maintained (> 500 bp), retained similar expression levels as in the monodirectional state [63, 66, 67]. In this work, the *P*_*HpFMD*_ and *P*_*HpMOX*_ promoters maintained their typical length and were not drastically shortened for the generation of the BDP fusion [62]. Hence, transcriptional spill over would be unlikely to occur between *P*_*HpFMD*_ and *P*_*HpMOX*_ considering the long 5′ regions in between. The *P*_*HpFMD*_ and the *P*_*HpMOX*_ promoters in addition to being strongly inducible by methanol also allow heterologous gene expression upon de-repression in the presence of growth limiting concentrations of carbon sources like glucose and glycerol [62]. This feature can be advantageous for many industrial fermentation facilities that are not certified for the use of large volumes of flammable solvents such as methanol.

Starting with HAC1 and PDI, different parts of the secretion machinery were explored including the over-expression of isomerases and flavoproteins involved in disulfide bond formation (Fig. 2). Digital PCR was used to determine that the gene copy number for all strains was one, enabling appropriate comparison of the chaperone effect without the interference of the effect of different gene copy numbers. Digital PCR was also used to follow *AppA* and *PDI* transcripts during phytase production and to study the transcriptional profile of the BDP *P*_*HpFMD-HpMOX*_. An approximate tenfold increase in transcription was observed for the *P*_*HpFMD*_ compared to *P*_*HpMOX*_ evidenced by the transcript levels of A*ppA* and *PDI*. Based on this result, and previous reports showing that the expression of the gene of interest is in most cases the limiting factor for production and not the folding chaperone [63, 66, 67], we co-expressed *AppA* phytase under the stronger promoter *P*_*HpFMD*_ and the folding chaperone under the weaker promoter *P*_*HpMOX*_. The rationale was to maximise the yield of the secreted protein of interest, whereas the intracellularly produced, mostly ER- targeted folding helpers are constrained by the cellular machinery and excessive amounts of chaperones may impair the secretion apparatus. Using *P*_*HpMOX*_ for PDI (or HAC1) co-expression showed very high *AppA* expression comparable to expression from *P*_*AOX1*_. We hypothesise that switching to *P*_*HpFMD*_ would give extremely high and unnecessary chaperone levels whilst reducing the amount of *AppA* by using the weaker *P*_*HpMOX*_ for its expression. Constitutive activation of the UPR through overexpression of HAC1 has been shown to enhance production of heterologous proteins in several systems including *S. cerevisiae*, *P. pastoris*, *A. niger* and mammalian cells [3, 22, 25, 59, 59, 60, 60]. Results from this work show that co-expression of HAC1 also improves production of AppA (Fig. 1b, Table 1). This regulator is most likely promoting secretion of the phytase by alleviating ER stress through the activation of UPR genes.

The co-expression of PDI improved production of AppA phytase in a similar way to HAC1. The isomerase might be important for the correct formation of the four disulfide bonds present in AppA structure, particularly for the non-consecutive bond. Yet, the expression of both HAC1 and PDI together did not have a synergistic effect in production of the phytase (Table 1). A possibility could be that the expression of HAC1 in AppA-HAC1 strain is inducing expression of PDI to similar levels as the expression of PDI in AppA-PDI (*P*_*HpFMD-HpMOX*_) strain. This hypothesis could also explain the inability to increase production of the phytase when co-expressing HAC1 in the AppA-PDI-HAC1 strain and the decrease of production in AppA-HAC1-PDI (Fig. 4d, Table 1). In fact, results obtained for the AppA-HAC1-PDI (*P*_*GAP*_) strain showed that expression of PDI from *P*_*GAP*_ was detrimental for phytase production, supporting the idea of fine-tunning and cumulative amounts as key factors determining success of multiple proteins co-expression [63, 66, 67].

The co-expression of the flavoproteins ERO1 and ERV2 was also investigated. ERO1 has been co-expressed with recombinant proteins in yeast improving the expression of human antibody Fab fragment in *S. cerevisiae*, while having no effect on the expression of trypsinogen *P. pastoris* [14, 23]. Due to the intrinsic feedback regulation of ERO1 and shuffling of disulfide bonds through cysteine pairs within the ERO1 structure proposed by Sevier et al. the overexpression of ERO1 without over-expression of PDI might not directly contribute to disulfide bond formation [14]. Results obtained for the AppA-ERO1 strain support this idea. Phytase production was not improved by sole co-expression of ERO1, while the co-expression PDI and ERO1 (from _*PGAP*_) slightly improved phytase production by 1.21 ± 0.06-fold (Fig. 4a, Table 1). Co-expression of PDI and ERO1 have been shown to increase the yields of lipase in *P. pastoris* [51].

The ERV2 flavoprotein has been characterised as an alternative pathway for disulfide bond formation in *S. cerevisiae*. ERV2 transfers oxidising equivalents to PDI by a dithiol–disulphide exchange reaction in a similar way as that of ERO1-dependent pathway [10, 24, 50, 58]. Gerber et al. reported that ERV2 can also directly oxidise substrate proteins due to the flexibility of the C-terminal arm containing a C-G-C motif that is responsible for disulfide exchange [24]. The putative membrane associated ERV2 flavoprotein from *P. pastoris*, which has 44% identity to ERV2 from *S. cerevisiae*, has not yet been characterised. Results from this work suggest that ERV2 in *P. pastoris* may represent an alternative pathway to ERO1 as in *S. cerevisiae* and may perhaps directly catalyse the formation of disulfide bonds as proposed by Gerber et al. [24]. The AppA-ERV2 (*P*_*HpFMD-HpMOX*_) strain presents similar phytase production to the AppA-PDI (*P*_*HpFMD-HpMOX*_) strain, and co-expression of PDI and ERV2 (under *P*_*GAP*_) further enhances phytase production by 1.35 ± 0.04-fold (Fig. 4a, Table 1). The capacity to re-establish reduced PDI to its oxidised state, either with ERO1 or ERV2, appears to be advantageous when over-expressing AppA to promote re-shuffling of possible incorrectly formed disulfide bonds. Further, the improvement in phytase secretion was dependent on the promotor used for the co-expression of ERO1 or ERV2. Only the strains where the flavoprotein was expressed from *P*_*GAP*_ showed improvement in phytase production. This observation could be related to timing the expression of the chaperones during growth and the requirement for flavoprotein availability ahead of phytase and PDI over-expression during glucose de-repression and methanol induction. On the other hand, the effect could be related to the strength of the promoter, where the expression driven by *P*_*CAT1*_ could potentially saturate the expression machinery and result in decreased AppA production when the cells are over-expressing two proteins (AppA phytase and PDI).

The formation of de novo disulfide bonds and isomerisation of incorrect ones can create a redox imbalance and generate reactive oxygen species (ROS) in the ER [14, 57]. The glutathione redox system controls redox homeostasis by regulating the glutathione redox ratio (GSH/GSSG). As part of this system, the cytosolic glutathione peroxidase GPX1 acts as an antioxidant enzyme that detoxifies ROS at the expense of reduced glutathione [14]. The over-expression of this enzyme has shown to improve the production of recombinant proteins in *P. pastoris* by increasing the oxidising environment of the ER [14]. Results presented here show that over-expression of GPX1 in AppA-PDI-GPX1 (*P*_*CAT1*_) strain slightly improve phytase production (Fig. 4c, Table 1). In this case, inducible expression of GPX1 under *P*_*CAT1*_ shows better results than constitutive expression under *P*_*GAP*_. The over-expression of GPX1 during growth could potentially create a redox imbalance in conditions where there is no folding oxidative stress.

Lastly, we also explored the co-expression of the vesicular transport proteins SLY1 and SEC1. Once heterologous proteins are correctly folded they can often be retained intracellularly resulting in poor secretion [31, 33]. However, the co-expression of SLY1 or SEC1 did not improve production of AppA phytase (Additional file 2: Fig. S1, Table 1). The limited AppA production in the AppA-SEC1 and AppA-SLY1 strains might still be related to incorrect folding and disulfide bond formation in the ER. Assessing phytase production in strains co-expressing PDI and SEC1 or SLY1 could be used to investigate whether a posterior trafficking bottleneck affects secretion of the phytase and limits productivity, however strains were not constructed in this work to test this hypothesis.

## Conclusions

The work presented here explores a wide variety of strain engineering possibilities for optimising protein expression in *P. pastoris* using *E. coli* AppA phytase as an example of a challenging protein of industrial importance. Multiple strategies to improve phytase production were tested since a single strategy does not exist to improve secretion of recombinant proteins.

This work used the rational design of strains to improve secretion of AppA, building on previous investigations and bringing new insights into secretion bottlenecks. Transcriptional optimisation revealed that *P*_*HpFMD*_ was the best promoter for phytase expression, improving yields by ~ 1.5- fold compared to *P*_*AOX1*_*.* Co-expression with HAC1 or PDI further improved yields by ~ 1.4-fold compared to the expression without chaperone. Finally, co-expression of a second chaperone, ERV2, further increased yields by ~ 2.0-fold, achieving a total synergistic improvement of ~ 3.0-fold compared to the initial yields obtained with *P*_*AOX1*_*.* Future work can now be performed to understand if the same production profiles would be observed for each strain under industrial fermentation conditions. The use of newly available tools, like BDPs, shows that protein production improvements can readily be achieved compared to the commonly used *P*_*AOX1*_ promoter and that there are a range of possibilities for further development of this important and widely used yeast microbial factory.

## Supplementary Information

**Additional file 1: Table S1.** Additional tables.

**Additional file 2: Fig. S1.** SEC1* or* SLY1 co-expression does not further improve phytase yields. Phytase expression was determined by the p-NPP assay after methanol induction. The AppA (*P*_*HpFMD*_) strain was included for comparison. Data are represented as mean values ± standard deviation (n = 3).

## Data Availability

The datasets used and/or analysed during the current study are available from the corresponding author on reasonable request.
