# Supplementary figures and images for: Synergistic optimisation of expression, folding, and secretion improves E. coli AppA phytase production in Pichia pastoris
Source: Microb Cell Fact. 2021 Jan 7;20:8. doi: 10.1186/s12934-020-01499-7 (PMC7836175; doi:10.1186/s12934-020-01499-7)

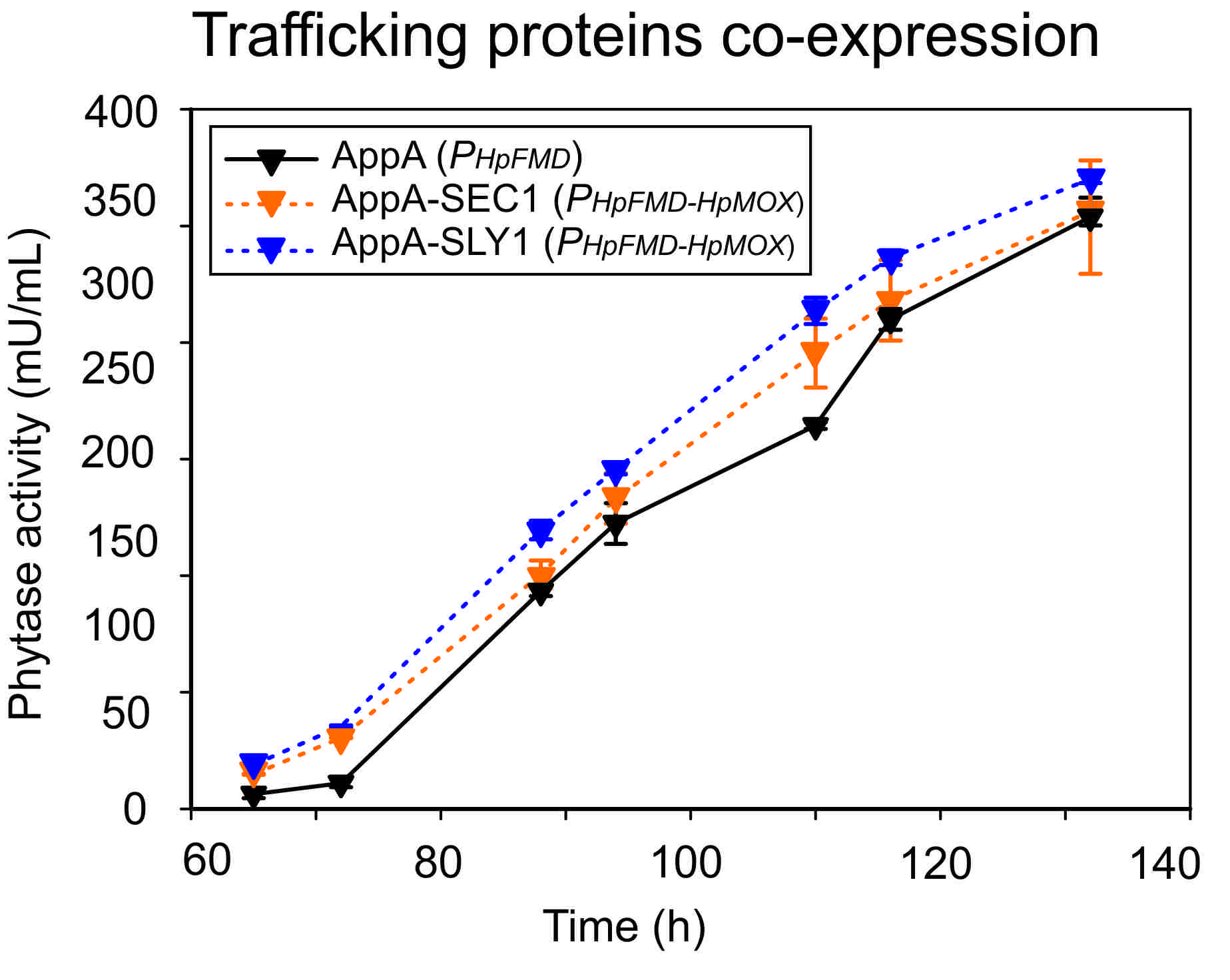

Supplement: Supplementary file 2 — Additional file 2: Fig. S1. SEC1 or SLY1 co-expression does not further improve phytase yields. Phytase expression was determined by the p-NPP assay after methanol induction. The AppA (PHpFMD) strain was included for comparison. Data are represented as mean values ± standard deviation (n = 3). [file 12934_2020_1499_MOESM2_ESM.jpg]
